# Study of serum uric acid and creatinine in hypertensive disorders of pregnancy

**DOI:** 10.5455/ijmsph.2015.15042015294

**Published:** 2015

**Authors:** Sapna Vyakaranam, Aparna Varma Bhongir, Dakshayani Patlolla, Rekha Chintapally

**Affiliations:** 1 Department of Biochemistry, MediCiti Institute of Medical Sciences, Ghanpur, Medchal Mandal Ranga, Reddy District, Telangana, India; 2 Department of Gynecology & Obstetrics, MediCiti Institute of Medical Sciences, Ghanpur, Medchal Mandal Ranga, Reddy District, Telangana, India

**Keywords:** Uric acid, creatinine, preeclampsia, gestational hypertension

## Abstract

**Background:**

Renal dysfunction, increased xanthine oxidase activity and oxidative stress in placenta contributes to the elevated uric acid levels in preeclampsia (PE).

**Objective:**

To determine serum uric acid and creatinine in hypertensive disorders of pregnancy and correlate with fetal outcome.

**Materials and Methods:**

Pregnant women ≥32 weeks of gestation. Study population included 3 groups, 31 normotensive pregnant (NP) women as controls, 30 pregnant women with gestational hypertension (GH) and 30 with PE.

**Result:**

Serum uric acid and creatinine levels were significantly elevated in PE (6.26±1.19 and 0.94±0.26 mg/dL) when compared with Pregnancy induced hypertension (PIH) (4.27± 1.0 and 0.66 ±0.19 mg/dL) and NP (4.25 ± 0.8 and 0.63± 0.13 mg/dL) (*P*-value <0.001 and <0.001) respectively. Receiver operation characteristics curves demonstrated greater sensitivity and specificity for uric acid (86.7% and 83.9%, respectively) in PE than for creatinine (80% and 77.4%, respectively). Uric acid had strong and negative correlation with fetal birth weight in PE (*r* = −0.59, *P* = 0.006), where as creatinine had negative but weak correlation (*r*= −0.03, *P*=0.87).

**Conclusion:**

Serum uric acid is a better diagnostic and predictive marker for PE and fetal outcome respectively.

## Introduction

Hypertensive disorders complicating pregnancy are the most common and serious medical disorder and constitute up to 2–10% of all pregnancies.^[1]^ Gestational hypertension (GH), preeclampsia (PE), and eclampsia are a part of a spectrum of hypertensive disorders that complicate pregnancy as specified by the National High Blood Pressure Education Program (NHBPEP) working group.^[2]^ Though studies have mentioned various parameters in etiopathogenesis of hypertensive disorders of pregnancy, still it remains obscure.

Serum uric acid and creatinine levels are a part of work up for the pregnant women with hypertension. The elevated levels of these parameters were due to decreased urinary clearance secondary to reduced GFR and increased reabsorption.^[3]^ Serum uric acid is not only a marker of severity of disease but also contributes to the pathology of disorder.^[4]^

Various studies have reported elevated levels of serum uric acid and creatinine in hypertensive disorders of pregnancy and also its effects on maternal and fetal outcomes.^[5–7]^ Very few studies have given an optimum cutoff for the both in predicting hypertensive disorders of pregnancy.^[6,8]^

The primary purpose of this study is to derive a cutoff for serum uric acid and creatinine in predicting hypertensive disorders of pregnancy (GH and PE) and also to look for its effect on fetal birth weight.

## Materials and Methods

This is a prospective study carried out in MediCiti Institute of Medical Sciences. Pregnant women with gestational age above 32 weeks, attending the antenatal clinic for regular checkups in department of obstetrics were enrolled in this study. The study populations were divided into 3 groups, 30 pregnant women diagnosed as having GH, 30 women has PE, and 31 normotensive pregnant women (NP) were considered as controls. All the participants were age matched. GH and PE were defined according to the International Society for the Study of Hypertension in Pregnancy (ISSHP).

GH is defined as denovo hypertension with systolic blood pressure (SBP) ≥140 mmHg and diastolic blood pressure (DBP) ≥90 mmHg after 20 weeks of gestation.

PE is GH with proteinuria – 1+ on dipstick or ≥300 mg/day or Pr:Cr ratio as ≥3.0 mg/g.^[2]^

Pregnant women with recurrent abortions, bad obstetric history, twins, preexisting medical disorders – such as diabetes mellitus, essential hypertension, renal disorders, cardiovascular, thyroid disorders, and liver disease – were excluded from the study. A written informed consent was obtained from women agreeing to participate in the study. The institutional ethics committee clearance was obtained. All the cases were followed until the delivery for maternal and fetal outcomes.

Blood pressure (BP) was measured by oscillometric digital sphygmomanometer (HEM-780N3; Omron, Made in Japan). Two measurements were taken 4 h apart. ISSHP guidelines were followed to measure BP.

Under strict aseptic conditions, 5 mL of blood sample was collected from all the participants by venous puncture, into properly labeled plain polystyrene tubes. For urine protein analysis, 10 mL mid stream urine was collected. Samples were collected, handled, and transported to the lab according to the guidelines given by clinical and laboratory standards institute/National Clinical Chemistry Laboratory Standards.^[9,10]^ Blood samples were centrifuged at 10,000 rpm for 10 min and the serum was separated. Serum uric acid and creatinine were estimated immediately on Dade Behring Dimension Xpand plus autoanalyser.

Serum uric acid was measured by modified uricase method.^[11]^ The normal serum reference range for females was 2.6–6.0 mg%. Serum creatinine was estimated by modified kinetic Jaffes method.^[12]^ The normal serum reference range for females was 0.6–1.0 mg%. Biorad QC level 1 and level 2 were run daily, the inter assay coefficient of variance (CV) for uric acid with assigned values of 4.89 and 9.03 mg/dL was 3.9 and 4.2%, respectively. The interassay CV for creatinine with assigned values of 2.16 and 6.39 mg/dL was 2.9% and 3.6%, respectively.

Urine proteins were analyzed by semiquantitative dipstick method. Two readings of 1+ (30 mg %) was considered as preeclampsia. All the participants were followed till delivery for maternal and fetal outcome. The weight of the newborn was recorded on digital weighing scale (SECA 354/364).

### Statistical analysis

The data were processed on MS excel work sheet and analysis will be carried out using MedCalc Statistical Software version 12.7.8 (MedCalc Software bvba, Ostend, Belgium; http://www.medcalc.org; 2014). The intergroup analysis was carried out by ANOVA and post hoc analysis. The critical values for maximum sensitivity and specificity were done on receiver operation characteristics (ROC) curves. The association between various parameters in a group was evaluated using Pearson’s correlation coefficient. A 2-tailed probability value of 0.05 was considered as statistically significant.

## Results

The study population included 31 NP women, 30 with GH, and 30 with PE. The demographic characteristics, laboratory parameters, maternal and fetal outcome are represented in Table 1. The mean age and gestational age among the three groups were not statistically significant. SBP, DBP, and urinary proteins were significantly different in the three groups.

The mean serum uric acid and creatinine levels were significantly elevated in PE (6.26 ± 1.19 mg/dL; 0.94 ± 0.26 mg/dL) when compared with GH (4.27 ± 1.0 mg/dL; 0.66± 0.19 mg/dL) and NP (4.25 ± 0.8 mg/dL; 0.63 ± 0.13 mg/dL), respectively.

In NP group, 67% women had full-term normal vaginal delivery (FTNVD) whereas it was 36.7% and 13.4% in PIH and PE groups, respectively. The fetal birth weight was significantly low in PE (2.31 ± 0.5 kg) when compared with NP (2.74 ± 0.58 kg) and PIH (2.8 ± 0.28 kg) groups.

The specificity and sensitivity of serum uric acid and creatinine in GH and PE are represented in Table 2. In ROC curve analysis, serum uric acid and creatinine had better sensitivity and specificity for PE when compared with GH.

**Figure 1:**
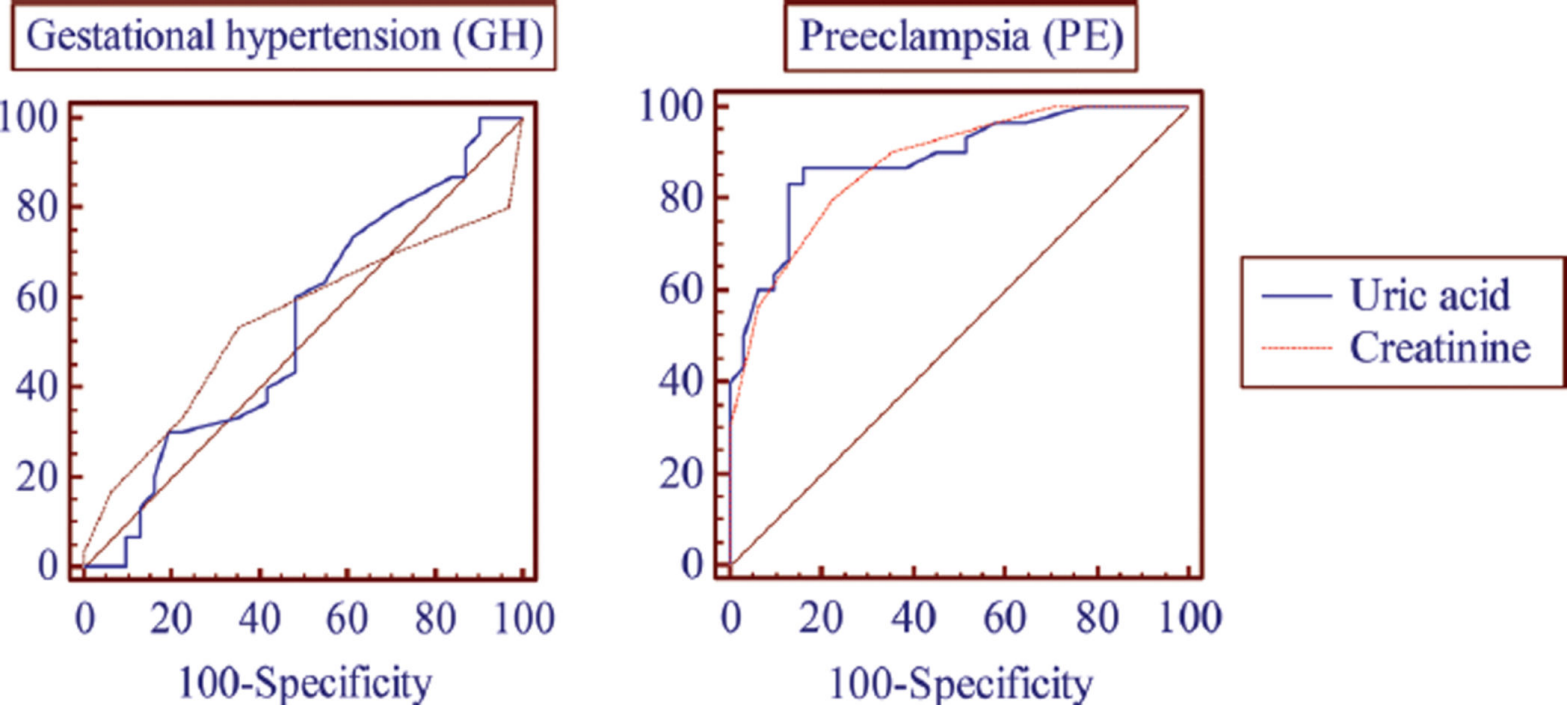
Comparison of receiver operation characteristics curves of serum creatinine and uric acid in gestational hypertension (GH) and preeclampsia (PE).

Comparison of ROC curves of serum uric acid and creatinine in GH and PE are represented in Figure 1. Serum uric acid and creatinine had significant Area Under Curve (AUC) 0.87 and 0.885, respectively, in PE when compared with GH 0.544 and 0.536, respectively.

**Figure 2:**
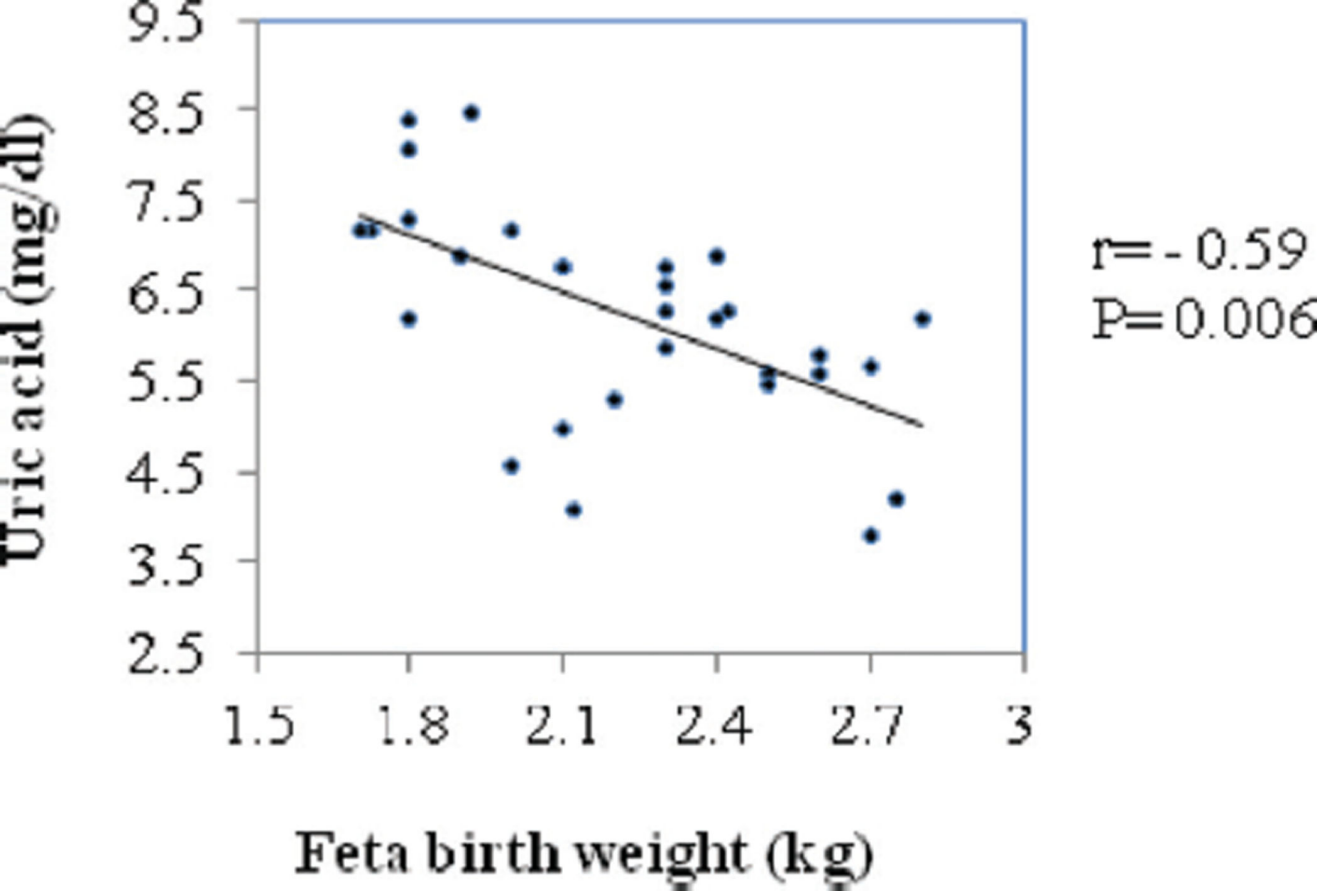
Correlation between fetal birth weight and serum uric acid in preeclampsia.

**Figure 3:**
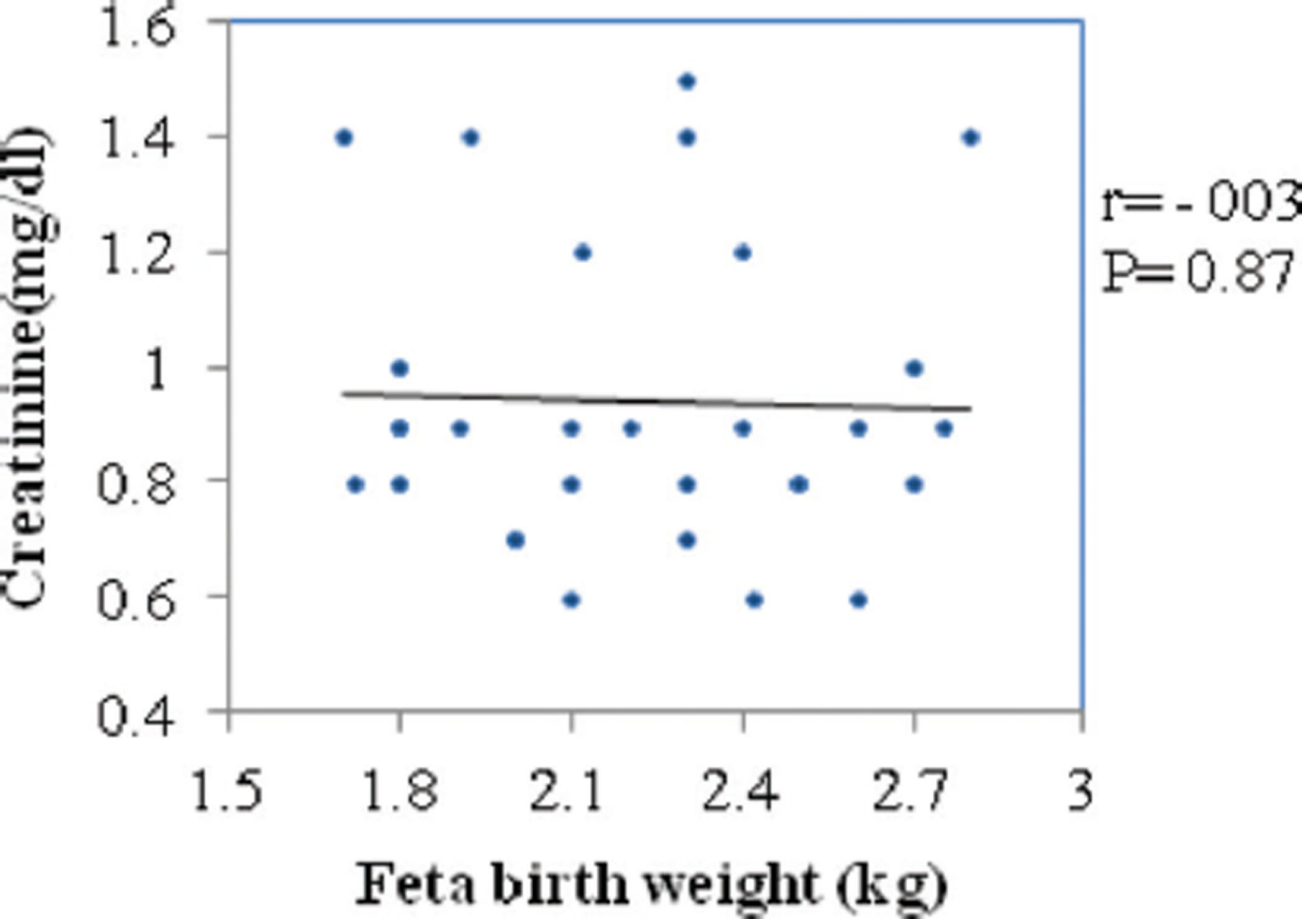
Correlation between fetal birth weight and serum creatinine in preeclampsia.

Correlation between fetal birth weight with serum uric acid and creatinine in PE are represented in Figures 2 and 3, respectively. Serum uric acid had significantly strong and negative correlation (*r* = −0.59; *P* = 0.006) with fetal birth weight whereas creatinine had negative but weak correlation (*r* = −0.03; *P* = 0.87) and was not statistically significant.

## Discussion

Hypertensive disorders of pregnancy are GH and PE, increase obstetrics risk, such as abruption placenta, preterm labor, eclampsia, and HELLP syndrome. Renal dysfunction in these disorders is due to glomerular endothelial injury causing decrease in GFR. Various studies have mentioned elevated levels of renal markers, such as serum uric acid, creatinine, and urea in PE.^[5–7]^

In our study, we observed a significantly elevated serum uric acid (6.26 ± 1.9 mg/dL) in PE. This is in accordance with the studies carried out by Padma et al. (6.13 ± 1.8 mg/dL)^[6]^ and Taefi et al. (5.8 ± 2.0 mg/dL)^[8]^ at 36 weeks of gestation. Uric acid is a metabolite of purine catabolism. Bainbridge and Roberts^[4]^ suggested that hyperuricemia in PE is multifactorial. In PE, elevated levels of uric acid are not only attributed to decreased renal excretion but also to increased oxidative stress resulting from placental ischemia and xanthine oxidase activity.^[13]^ In our study no significant difference in serum uric acid was observed between GH and NP.^[6]^

In ROC analysis a cutoff value of 4.9 mg/dL had 86.7% sensitivity and 83.9% specificity for serum uric acid. Padma et al.^[6]^ in their study observed sensitivity of 75% and specificity of 76.8% with a cutoff of 5.0 mg%. Powers et al.^[14]^ in their study observed an early elevation of serum uric acid, that is, at 10 weeks gestation in women who later developed PE.

The mean fetal weight was significantly low in PE (2.31 ± 0.5 kg) when compared with GH and NP. This is in agreement with the study carried out by Padma et al. (2.3 ± 0.8 kg).^[6]^ In our study, we found uric acid had significant negative and strong correlation with fetal birth weight in PE. Hawkins et al.^[15]^ in a retrospective cohort demonstrated that increasing level rates of uric acid beyond 5.9 mg/dL are associated with adverse fetal outcome such as small for gestation age and preterm birth. As placental hypoxia contributes to the elevated levels of uric acid in PE, it acts as a better marker for hypoxia of fetal outcomes than maternal distress.^[16]^ Hawkins et al.^[15]^ and Livingston et al.^[17]^ concluded that uric acid is clinically useful in predicting fetal outcome than maternal.

Serum creatinine is a marker of GFR and renal dysfunction. In our study, we observed elevated levels of serum creatinine in PE when compared with GH and NP. A cutoff of 0.7 mg/dL of serum creatinine had 80% sensitivity and 77.4% specificity. This is in accordance with previous studies.^[6]^ In our study, we did not observe significant correlation between creatinine and fetal weight, indicating its limited role in predicting fetal outcome.

## Conclusion

Serum uric acid and creatinine are elevated in PE whereas no significant difference was observed between GH and NP. Serum uric acid had better specificity and sensitivity for PE and also correlated negatively with fetal birth weight. Serum uric acid and creatinine levels vary with gestational age, hence studies should focus on estimating these markers in all trimesters so as to diagnose PE at an early stage and avert maternal and fetal outcome. Small sample size is the limitation of our study.

## Figures and Tables

**Table 1:** Demographic characteristics, laboratory parameters, maternal and fetal outcomes in study groups

Parameter	NP (*n* = 31)	GH (*n* = 30)	PE (*n* = 30)	*P*-Value
Age (years)	23.2 ± 2.9	22.9 ± 3.0	23.7 ± 3.4	0.582
Gestation age (weeks)	36.4 ± 3.5	36.9 ± 2.9	35.8 ± 2.6	0.373
SBP (mmHg)	113.2 ± 7.5	143.3 ± 8.0	148.3 ± 9.9	<0.001*
DBP (mmHg)	75.2 ± 7.7	96.0 ± 7.2	103.0 ± 11.5	<0.001*
Serum uric acid (mg/dL)	4.25 ± 0.80	4.27 ± 1.0	6.26 ± 1.19	<0.001*
Serum creatinine (mg/dL)	0.63 ± 0.13	0.66 ± 0.19	0.94 ± 0.26	<0.001*
Urine proteins				<0.001*
Nil	30	23	0	
Trace	1	7	1	
1+			12	
2+			10	
3+ and above			7	
Type of delivery				<0.001*
No. of vaginal deliveries	21	11	4	
No. of cesarean sections	10	19	26	
Birth weight (kgs)	2.8 ± 0.28	2.74 ± 0.58	2.31 ± 0.5	<0.001*

NP, Normal pregnancy; GH, gestational hypertension; PE, preeclampsia.

* Significant *P*-value <0.05.

**Table 2:** Specificity and sensitivity of serum uric acid and creatinine in GH and PE

	Cutoff	Sensitivity (%)	Specificity (%)	AUC	95% CI
GH					
Uric acid (mg%)	≤3.9	36.7	58.1	0.536	0.404–0.665
Creatinine (mg%)	>0.6	70	29.9	0.544	0.412–0.672
PE					
Uric acid (mg%)	>4.9	86.7	83.9	0.885	0.778–0.953
Creatinine (mg%)	>0.7	80	77.4	0.87	0.766–0.946

GH, gestational hypertension; PE, preeclampsia
